# Trends and prevalence of overweight and obesity in primary school aged children in the Republic of Ireland from 2002-2012: a systematic review

**DOI:** 10.1186/1471-2458-14-974

**Published:** 2014-10-14

**Authors:** Eimear Keane, Patricia M Kearney, Ivan J Perry, Cecily C Kelleher, Janas M Harrington

**Affiliations:** Department of Epidemiology and Public Health, University College Cork, Fourth Floor, Western Gateway Building, Western Road, Cork, Republic of Ireland; School of Public Health, Physiotherapy and Population Science, University College Dublin, Dublin, Republic of Ireland

**Keywords:** Ireland, Prevalence, Trends, Obesity, Children

## Abstract

**Background:**

The prevalence of childhood overweight and obesity in developed countries appears to be levelling off. As trends in childhood obesity prevalence have not been examined over the past decade in the Republic of Ireland, this systematic review aims to compile and synthesise all available information on the prevalence of overweight and obesity in primary school aged children between 2002 and 2012.

**Methods:**

Systematic review of published and grey literature containing data on objectively measured height and weight. Inclusion criteria included studies where data was collected between 2002 and 2012 from at least 200 primary school aged children in the Republic of Ireland. Database searching, Google searching, reference searching and contact with obesity experts was undertaken. Overweight, obesity and morbid obesity were defined using standard International Obesity Taskforce definitions. Study quality was assessed.

**Results:**

Fourteen studies (16 prevalence estimates) met the inclusion criteria. The combined prevalence of overweight and obesity within the studies ranged from 20-34%. No significant trend in overweight prevalence over time was observed (p=0.6). However, there was evidence of a slight decrease in obesity prevalence over the period (p=0.01), with a similar though non-significant decline in the prevalence of morbid obesity (p=0.2).

**Conclusion:**

The findings of this systematic review require cautious interpretation though the prevalence of childhood overweight and obesity in the Republic of Ireland has reached a plateau and may be falling. These findings provide some ground for optimism though the current plateau is at an unacceptably high level. Thus current population based preventive strategies need to be sustained and intensified.

**Electronic supplementary material:**

The online version of this article (doi:10.1186/1471-2458-14-974) contains supplementary material, which is available to authorized users.

## Background

Globally, childhood overweight and obesity is a significant public health problem associated with a number of adverse physical and psychological consequences during childhood and in later life [1–3]. In the latter three decades of the 20^th^ century, a two to three fold increase in overweight and obesity prevalence in school aged children was reported in many industrialised regions including countries in North America and Western Europe [4]. By the year 2000, estimates suggested that between 25-33% of all children in many developed countries were either overweight or obese [5, 6] and future projections anticipated prevalence rates would continue to increase significantly [7].

However, recent evidence from some developed countries suggests that childhood overweight and obesity prevalence rates have stabilised since the early 2000s [8, 9]. Olds et al. [10] collated data from 467,294 children from 9 countries (including countries from Western Europe, North America, Oceania and Asia) and separately assessed overweight and obesity trends over time. The authors found that trends in both overweight and obesity prevalence appeared to be stabilising between 1995 and 2008. Rokholm et al. [11] conducted a systematic review and assessed the prevalence of childhood obesity in 17 countries (including countries in Western Europe, North America and Australia) since the year 1999. While there was some conflicting evidence, overall the findings suggested that obesity prevalence had stabilised in many developed countries though patterns were less consistent amongst lower socio-economic groupings.

Perry et al. [12] collated data from three large scale national surveys on the height and weight of Irish children between 1948 and 2002. The findings indicated that the weight of Irish children had increased disproportionally to their height. In 2008, the World Health Organisation (WHO) childhood obesity surveillance initiative commenced in Ireland and this initiative will provide ongoing data on the height and weight of Irish children aged 7 [13]. However, trends in childhood overweight and obesity in the Republic of Ireland (ROI) have not been examined over the past decade. The prevalence of morbid obesity in Irish children also remains unknown. Therefore, this systematic review aims to objectively synthesise all available information on the prevalence of overweight and obesity (including morbid obesity) in primary school aged children in the ROI over a ten year period from 2002-2012.

## Methods

### Search strategy

**Figure 1:**
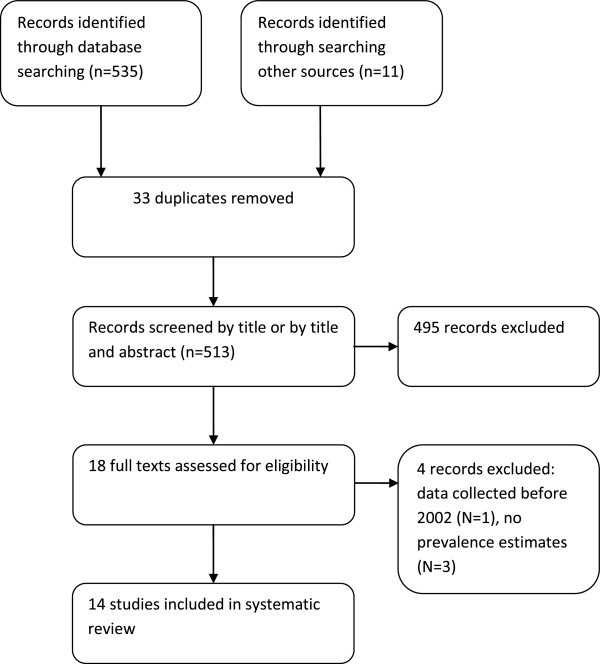
**Flowchart of studies included in the review.**

The search strategy is summarised in Figure 1 with further details available in Additional file 1. Medline, EMBASE, Academic search complete and CINAHL were systematically searched for relevant literature in April and May 2013. For each database, searching was conducted using a combination of the following search terms: obesity, overweight, obese, body mass index, BMI, Ireland, Irish, child*, school children, schoolchildren, pediatr*, paediar*, girls, boys, prevalence, rate, trend, increase, decrease. Search terms were combined using the AND or OR operators. Limits were applied on year of publication (from 2002 onwards) and age (primary school age) of participants.

A Google search was conducted in May 2013 using the search terms: prevalence, child, obesity, Ireland. Google advanced search commands were applied using the ‘site or domain’ option with .ie webpage’s searched only. The first 20 pages were searched for relevant literature. Publically available Irish databases or national agencies websites (Irish Social Science Data Archive, Safefood, The Health Well, Department of Health and Children’s Irish child health database) known to the authors of this review and available on the Internet were searched for relevant literature in April and May 2013. A number of obesity experts working in Ireland were identified by the authors of this systematic review. Each expert was contacted either by email or via an announcement made at an Irish obesity action meeting held in June 2013 (http://www.safefood.eu/Professional/Nutrition/All-island-Obesity-Action-Forum.aspx). Information was sought on any data sources not located during the database searching. Data sources known to the authors of this review were also considered for inclusion. A reference search of all eligible papers was conducted to identify additional literature. Findings from one included study (the WHO European Childhood Obesity Surveillance programme) were updated during the writing of the review and the updated findings included in the current review [14].

### Inclusion criteria

Inclusion criteria for this review were as follows:

1. Studies conducted in the ROI where data collection was undertaken between 2002-2012;
2. Cross-sectional or cohort studies where height and weight were objectively measured;
3. Studies reporting overweight and obesity prevalence estimates using International Obesity Taskforce (IOTF) [15] definitions for body mass index (BMI) or where data was available to calculate BMI;
4. Studies including at least 200 children of a primary school age (approximately 4-12 years).

Peer-reviewed publications, grey literature and baseline data from population based intervention studies were considered for inclusion. Studies containing participants from Northern Ireland only, self-reported data or which reported the effect of a treatment or intervention for childhood obesity were excluded.

### Quality assessment and data extraction

The methodological quality of all included studies was assessed and extracted by two independent reviewers (EK, JMH). Any disagreements were resolved by consensus. Additional file 2 provides an outline of the quality assessment criteria. Eight criteria were used which were adapted from those outlined by Radulescu et al., 2009 [16] for assessing the quality of prevalence studies. The quality of included papers were categorized as ‘high’ if 7-8 criteria were met, ‘moderate’ if 5-6 criteria were met and ‘low’ if 4 or less criteria were met.

### Statistical analysis

Data analysis for this review was conducted in Stata 12 IC (StataCorp LP, USA). Where we were provided with raw data, children were categorised using the zbmicat function (a Stata add-on program) as normal weight, overweight or obese using age and gender specific IOTF definitions [17]. Year of data collection was ranked from oldest to newest and Cuzick’s non-parametric trends test was used to conservatively test for trends in overweight and obesity prevalence over time. Trends were assessed separately for all studies, nationally based and regionally based studies. Within the included studies, trends in overweight and obesity over time were assessed separately for girls and boys. The included studies were grouped into 3 independent categories based on the age range of the participating children as 4-7.9 years only, 8-13.9 year only or 4-13.9 years. Trends in overweight and obesity were then assessed separately within each of the age groups. We had access to raw data from three included studies, [18] [Keane et al., unpublished observations] [HSE Meath, unpublished observations] to estimate the prevalence of morbid obesity (BMI cut-off of 35 km/m^2^) using extended IOTF definitions [19]. A fourth study with available data was excluded as height and weight measures were truncated [20]. Children were classified as morbidly obese based on gender and 6 month age category.

## Results

### Identification and selection of studies

Five hundred and thirty five titles were retrieved from electronic database searching and 11 from the other sources searched. Duplicate titles were removed (N = 33) and 513 titles/abstracts were reviewed and considered for inclusion. After initial screening of titles and abstracts, 19 full texts were retrieved and read for relevance. Electronic database searching resulted in 8 studies being identified for inclusion, of which one study was updated during the writing of this systematic review. One further relevant study was identified during reference searching, 3 from contact with obesity experts and 2 from the authors of this reviews awareness of other grey literature sources. Overall, 14 studies (with 16 prevalence estimates reported in 15 papers) met all the inclusion criteria. Figure 1 displays the results of the search strategy.

### Description of included studies

Table 1 describes each of the included studies. The included studies were primarily cross-sectional. One study was a retrospective cohort study and two studies were baseline findings from intervention studies. Four studies (6 prevalence estimates) were based on national samples whereas 10 were regional samples. The sample sizes ranged from 204 to 14,036. Table 2 contains details on the methods of measurement and the limitations (which were identified by the authors of this review) of each study. Of the included studies, 5 studies were considered to be of ‘high’ quality, 9 of ‘moderate’ quality and 1 of ‘low’ quality. Additional file 2 contains details on the critical appraisal of each included study. Overall, the combined prevalence of overweight and obesity in the national and regional studies ranged from 20-26% and 21-34% respectively.

**Table 1:** **Descriptive information of included studies**

Author	Data collection years	Estimating prevalence primary aim of study	Sample size#	National or regional data	Age	Setting	Response rate	Design	Sampling	Study quality (out of 8)
**Nationally based data**										
Whelton et al. [21]	2001-2002	Yes	14036	National	4-13	Primary schools	68% of children	Cross-sectional	Clustered sampling with schools as the clustering unit. Children were randomly selected on the basis of age, gender, location of school and water type. Primary school children in junior infants, second and sixth class (year 1, 4 & 8 of enrolment) were invited to take part	6
O’Neill et al. [22]	2003-2004	Yes	596	National	5-12	Primary schools	66% of children	Cross-sectional	A list of primary schools was obtained from the Dept of Education and Science. Schools were categorised by location, gender, size and disadvantaged status. Schools were randomly selected from each category and children randomly selected and invited to take part	5
Layte & McCrory [23]	2007-2008	Yes	8136	National	9.0-9.9	Home	57% of children	Cross-sectional analysis of a longitudinal study	In stage one, primary schools were randomly selected using a probability proportionate to size (PPS) sampling method and in stage two a random sample of age eligible children from within each school were invited to take part	7
Heavey et al. [13]	2008	Yes	2420	National	7.0-7.9	Primary schools	72% of children	Cross-sectional, round 1 of WHO COSI programme	A nationally representative sample of primary schools were selected using a PPS sampling strategy. Children in first class (year 3 of enrolment) were recruited to participate. One class of first class children were selected from large schools	7
Heinen et al. [14]	2010	Yes	996	National	7.0-7.7	Primary schools	64% of children	Cross-sectional, round 2 of WHO COSI programme	Schools who took part in round 1 [12] of this surveillance initiative were invited to take part in round 2. Only children aged 7 in first class were considered in this current analysis. One class of first class children were selected from large schools	6
Heinen et al. [14]	2012	Yes	991	National	7.0-7.7	Primary schools	55% of children	Cross-sectional, round 3 of WHO COSI programme	Schools who took part in round 1 [12] of this surveillance initiative were invited to take part in round 3. Only children aged 7 in first class were considered in this current analysis. One class of first class children were selected from large schools	6
**Regionally based data**										
McMaster et al. [24]	2001-2002	Yes	328	Regional (Counties Leitrim and Cavan)	4.2-7.9	Primary schools	91% of records had height & weight measures	Retrospective cohort	All senior infants (year 2 of enrolment) from all schools in the former North Western Health Board area. Paper copies of school health records were retrospectively hand searched for height and weight data in March 2003	7
Harrison et al. [25]	2003	No	312	Regional (South-East of Ireland)	9-11	Primary schools	99% of children	Baseline findings from a health education intervention	Schools in areas of social disadvantage located in the South East of Ireland were recruited to participate and children from 4^th^ class (year 6 of enrolment) were invited to partake	5
Evans et al. [26]	2004-2007	Yes	3493	Regional (County Mayo)	6.0-6.9	Primary schools	99.7% of children	Cross-sectional	All children from all 189 primary schools in County Mayo had height and weight measures taken as part of the school health check between February 2005 and June 2008	7
Barron et al. [27]	2007	Yes	969	Regional (County Kildare)	4.5-13.5	Primary schools	83% of children	Cross-sectional	Data collected from 2 single sex primary schools in a town in County Kildare as part of a larger research project	5
Murrin et al. [28]	2007-2008	No	529 (at follow up)	Regional data (Counties Dublin and Galway)	5-7	Home	62% of mothers at follow-up	Cross-sectional analysis of a prospective observational cross-generational linkage cohort	Sample of 1124 expectant mothers recruited at 1^st^ antenatal hospital visit in 2 hospitals over an 18 month period from 2001-2003 [29]	7
Belton et al. [30]	2008	No	301	Regional (greater Dublin)	6-9	Primary schools	97% of children	Cross-sectional	Four mixed gender schools from the greater Dublin area were selected to take part in the study	3
Fitzgerald, [31]	2008-2009	No	204	Regional (West of Ireland)	9-12	Primary schools	58% of children	Cross-sectional	Primary schools were randomly selected from the Department of Education and Science list of schools and invited to take part in the study. All children in 4^th^ to 6^th^ class (years 6-8, of enrolment) were invited to take part	6
HSE Meath, 2009 (unpublished observations)	2009	Yes	1468	Regional (County Meath)	11-13	Primary schools	63% of children	Cross-sectional	A complete sample of primary schools from County Meath were invited to partake and all children in 6^th^ class (year 8 of enrolment) of participating schools invited to take part	6
Hollywood et al. [32]	2009	No	537	Regional (County Dublin)	4-12	Primary schools	Details not provided	Baseline findings from a prospective cohort study	Primary school children from urban disadvantaged areas located in Revitalising Areas by Planning Investment and Development (RAPID) areas in Dublin took part in study. All children in Junior infants to 5^th^ class (year 1- 7 of enrolment) were invited to take part	5
Keane et al. 2013 (unpublished observations)	2012-2013	Yes	1068	Regional (County Cork)	8-11	Primary schools	65% of children	Cross-sectional	A list of primary schools was obtained from the Dept of Education and Science website. Schools were recruited using a PPS sample (with further purposive sampling) of Cork city primary schools and all rural schools from one area in Cork County were invited to partake. All children in 3^rd^ and 4^th^ class (year 5 and 6 of enrolment) were invited to take part	6

#Sample sizes only include valid number of age eligible participants who provided valid objective height and weight measures.

**Table 2:** **Details on method of measurements and limitations of the included studies**

Author	Data collection year(s)	Height measure	Weight measure	Method of measurement	Measurement personnel	Limitations^
**Nationally based data**						
Whelton et al. [21]	2001-2002	Leicester portable height measure	Soehnle 7403 Mediscale	Height was measured to the nearest 1 decimal point in centimetres (cm) and weight to the nearest 1 decimal point in kilograms (kg). Shoes, heavy clothing and headgear were removed for measures	Trained researchers took measures using a standard protocol	Response rate not adequate and no information given on non-responders
O’Neill et al. [22]	2003-2004	SECA Leicester height measure	SECA 770 digital weight scales	Height was measured in the Frankfurt plane position to the last complete millimetre (mm) and weight to the nearest 0.1 kg. Light indoor clothing was worn for measures without shoes, hair ornaments, pony tails undone and empty pockets	Qualified nutritionists took measures	Response rate not adequate, no information given on non-responders and methods to reduce observer bias not outlined
Layte & McCrory, [23]	2007-2008	Leicester portable height measure	SECA 761 flat mechanic scales	Height was measured to the nearest mm and weight to the nearest 0.5 kg. Light clothing was worn for measures	Trained researchers took measures	Response rate not adequate and no information given on non-responders**
Heavey et al. [12]	2008	SECA 214 portable stadiometer	SECA 872 weighing scales	Height was measured to the last complete mm and weight to the nearest 0.1 kg. Light indoor clothing was worn for measures without shoes, hair ornaments, pony tails undone and empty pockets	Trained researchers took measures using a standard protocol	Response rate not adequate and no information given on non-responders
Heinen et al. [14]	2010	SECA 214 portable stadiometer	SECA 872 weighing scales	Height was measured to the last complete mm and weight to the nearest 0.1 kg. Light indoor clothing was worn for measures without shoes, hair ornaments, pony tails undone and empty pockets	Trained researchers took measures using a standard protocol	Response rate not adequate and no information given on non-responders
Heinen et al. [14]	2012	Leicester height measure	HD-305 Tanita weighing scales	Height was measured to the last complete mm and weight to the nearest 0.1 kg. Light indoor clothing was worn for measures without shoes, hair ornaments, pony tails undone and empty pockets	Trained researchers took measures using a standard protocol	Response rate not adequate and no information given on non-responders
**Regionally based data**						
McMaster et al. [24]	2001-2002	Leicester height measure	Hansen digital weight scales	Height measured to the nearest 0.5 cm and weight to the nearest 500 g. Light clothing was worn for measures without shoes, jackets and headgear	Two school nurses took measures using a standard protocol	No information given on non-responders
Harrison et al. [25]	2003	Seca Leicester height measure	Seca digital floor scales	Children wore light clothing, without shoes for measures	Researchers were trained in anthropometry	Sampling method unclear, no information given on non-responders and not enough detail provided on method of measurement
Evans et al. [26]	2004-2007	Leicester height measure	Tanita solar weight scales	Height was measured to the nearest 0.1 cm and weight to nearest 0.1 kg using a standard protocol [33]	Trained public health nurses took measures. Intra-observer variability was measured	No information given on non-responders
Barron et al. [27]	2007	Leicester height measure	Tanita WB-100 digital medical weighing scales	Children wore tracksuits, without shoes for measures	One qualified paediatric nurse took all measures	Sampling method used not clear, no information given on non-responders and not enough detail provided on method of measurement
Murrin et al. [28]	2007-2008	Leicester height measure	Tanita digital weight scales model HD305	Height was measured to the nearest 1 cm and weight to the nearest 0.1 kg. A standard protocol was used	Trained researchers took measures using standard procedures	Response rate not adequate
Belton et al. [30]	2008	SECA Leicester height measure	SECA heavy duty scales	No details given	No details given	Sampling method unclear, no information given on non-responders, height and weight measurements methods used not described, inadequate detail on equipment used and efforts to reduce observer bias not stated
Fitzgerald, 2010 [31]	2008-2009	Leicester height measure	Seca 899 weight scales	Height was measured to the nearest 0.1 cm in the Frankfurt plane position and weight to the nearest 0.1 kg. Measures were taken without heavy clothing and shoes	Standard procedures were used. Intra observer variability was tested	Response rate not adequate and no information given on non-responders
HSE Meath, 2009 (unpublished observations)	2009	Leicester height measure	Soehnle 7403 Mediscale	Height was measured in the Frankfurt plane position to the nearest 1 decimal point in cm and weight to the nearest 1 decimal point in kg. Measures were taken without shoes and without excessive clothing	Researchers trained prior to data collection. Inter examiner agreement was tested	Response rate not adequate and no information given on non-responders
Hollywood et al. [32]	2009	SECA Leicester portable height measure	SECA 875 digital flat scales	Height was measured in the Frankfurt plane position. Measures were taken in stockings without heavy outdoor clothing	One trained children’s nurse took all the measures	Sampling method unclear, response rate not adequate, no information given on non-responders and not enough detail provided on method of measurement
Keane et al. 2013 (unpublished observations)	2012-2013	Leicester portable height measure	Tanita WB100MA mechanic scales	Height was measured in the Frankfurt plane position to the nearest mm and weight to the nearest 0.1 kg. Measures were taken without shoes and in light clothing	Trained researchers took measures using standard procedures	Response rate not adequate and no information given on non-responders

**The data was probability weighted prior to analysis to account for the complex sampling design. This involved the structural adjustment of the study sample to the population level whilst maintaining the case base of participating children, ^ The limitations outlined in this table were identified by the authors of this systematic review during critical appraisal of each study.

### Prevalence of overweight and trends over time

**Figure 2:**
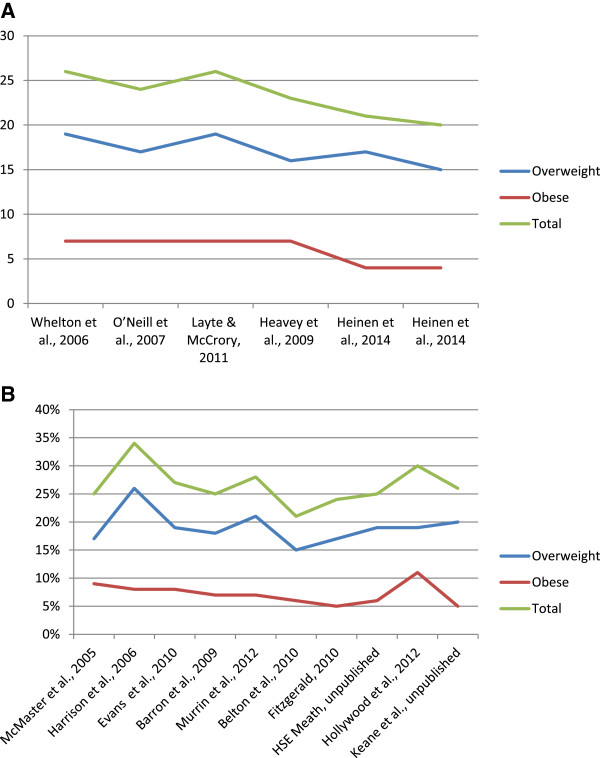
**Prevalence of childhood overweight and obesity within the (A) nationally and (B) regionally based studies.** Studies are presented by year of data collection. The study on the left represents the prevalence of overweight and obesity from the study which collected data least recently. The study which collected data most recently is presented on the right.

Figure 2 and table 3 describe the prevalence of overweight and obesity within each included study. Within the national and regional based studies, the prevalence of overweight ranged from 15-19% and 15-26% respectively. The prevalence of overweight ranged from 17-21%, 15-26% and 15% within the ‘high’, ‘moderate’ and ‘low’ quality studies. No significant trend in overweight prevalence was observed over time among all included studies (p=0.6), national studies (p=0.09) or regional studies (p=0.8).

**Table 3:** **Prevalence of overweight and obesity in the included studies** ^**#**^

Study	Data collection year(s)	Sample size	Age range	Prevalence of overweight			Prevalence of obesity (including morbid obesity)			Prevalence of overweight and obesity		
				Boys (%)	Girls (%)	Total (%)	Boys (%)	Girls (%)	Total (%)	Boys (%)	Girls (%)	Total (%)
**Nationally based data**												
Whelton et al.* [21]	2001-2002	14036	4-13	17%	21%	19%	6%	8%	7%	23%	29%	26%
O’Neill et al. [22]	2003-2004	596	5-12	15%	20%	17%	4%	9%	7%	19%	29%	24%
Layte & McCrory [23]	2007-2008	8136	9.0-9.9	17%	22%	19%	5%	8%	7%	22%	30%	26%
Heavey et al. [13]	2008	2420	7.0-7.9	13%	19%	16%	5%	8%	7%	18%	27%	23%
Heinen et al.^^ [14]	2010	1011	7.0-7.7	14%	20%	17%	4%	5%	4%	18%	24%	21%
Heinen et al.^^ [14]	2012	1002	7.0-7.7	14%	17%	15%	3%	5%	4%	17%	22%	20%
**Regionally based data**												
McMaster et al., 2005 [24]	2001-2002	328	4.2-7.9	16%	18%	17%	9%	8%	9%	25%	26%	25%
Harrison et al.^ [25]	2003	312	9-11	27%	24%	26%	7%	9%	8%	34%	33%	34%
Evans et al. [26]	2004-2007	3493	6.0-6.9	17%	22%	19%	6%	9%	8%	23%	31%	27%
Barron et al. [27]	2007	969	4.5-13.5	18%	18%	18%	7%	7%	7%	24%	25%	25%
Murrin et al. [28]	2007-2008	529	5-7	19%	23%	21%	7%	8%	7%	25%	30%	28%
Belton et al.^ [30]	2008	301	6-9	14%	15%	15%	6%	6%	6%	20%	21%	21%
Fitzgerald^ [31]	2008-2009	204	9-12.9	14%	24%	17%	9%	2%	5%	22%	26%	24%
HSE Meath, 2009 (unpublished observations)*	2009	1468	11-13	17%	20%	19%	4%	7%	6%	22%	28%	25%
Hollywood et al. [32]	2009	537	4-12	15%	23%	19%	12%	10%	11%	27%	33%	30%
Keane et al. (unpublished observations)*	2012-2013	1068	8-11	20%	21%	20%	4%	7%	5%	24%	28%	25%

^#^all prevalence estimates are rounded to the nearest whole number, as a result some numbers may appear not to add but this is due to rounding up or down of prevalence estimates, ^author of study contacted and asked to provide prevalence rates for overweight and obesity using IOTF definitions, ^^due to the complexity of the WHO European Childhood Obesity Surveillance programme data, only prevalence estimates from the 7 year olds is presented in this current review, *EK conducted the analysis to obtain prevalence estimates of overweight and obesity using IOTF definitions.

### Prevalence of obesity and trends over time

The prevalence of obesity ranged from 4-7% in the nationally based studies. The prevalence of obesity ranged from 5-11% in the regional studies. The prevalence of obesity ranged from 7-9%, 4-11% and 6% within the ‘high’, ‘moderate’ and ‘low’ quality studies. A small, significant declining trend in obesity prevalence was observed over time when all studies were reviewed (p=0.01). No significant trend over time was observed for the national (p=0.09) studies and a borderline significant trend over time was observed for the regional studies (p=0.05). When overweight and obesity prevalence rates were combined, trends were not significant.

### Prevalence of morbid obesity and trends over time

Morbid obesity prevalence estimates were available for three of the included studies. Based on year of data collection from least to most recently collected data, the prevalence of morbid obesity in each of the three studies was 2.2% [18], 1.0%, [Keane et al., unpublished observations] and 0.8% [HSE Meath, unpublished observations]. The highest prevalence estimate was reported in the earliest (2002) study. The reduction in estimates over time was not significant (p=0.2).

### Prevalence and trends by age and gender

The prevalence of overweight and obesity in the national studies was consistently higher in girls than boys. Within the included studies, a significant trend over time was observed for obesity rates in girls in all included studies (p=0.04) but not in boys (p=0.2). When trends in overweight and obesity prevalence over time were assessed within the studies that collected data in children aged 4-7.9 years only, 8-13.9 years only and from 4-13.9 years, no significant trends were observed.

## Discussion

### Main findings

This systematic review aimed to synthesize all available overweight and obesity prevalence data from primary school children in the ROI between 2002 and 2012. Fourteen studies (16 prevalence estimates) were included in the review. Due to limited comparability between studies, the results of this review were difficult to interpret. However, similar to trends in other developed countries [11, 34], this review suggests that while childhood overweight and obesity prevalence rates remain high in Ireland, prevalence rates appear to be stabilising.

Within the included studies, no trend in overweight prevalence was observed over time. Overweight prevalence varied slightly (non-significant trend) in the nationally based studies with the lowest prevalence of overweight reported in the study where data was collected most recently [14]. This may reflect the age of the included participants rather than a decrease in the prevalence of overweight. The children who participated in the most recent studies [13, 14] were 7 years of age. Pubertal maturation is associated with an increased BMI [5, 35] and this may partly explain the lower prevalence of overweight and obesity in the later completed study. Alternatively, differences in methodologies between studies may explain findings.

A significant trend over time in obesity prevalence was observed. Obesity prevalence remained constant at 7% in the nationally based studies between 2002 and 2008 with the prevalence of obesity reducing to 4% thereafter. The results from the regionally based studies were difficult to interpret and prevalence rates varied considerably between studies. The quality of some of the regional studies or the generalisability of the study populations may act as an explanation. For example, two of the regional studies [25, 32] were completed in areas of high social deprivation. Thus, higher prevalence rates may have been estimated in these studies as a lower socio-economic status is associated with an increased risk of obesity [36].

Morbid obesity data was available for three of the included studies. The results suggest that up to 1 in 50 Irish children are morbidly obese. The lower prevalence of morbid obesity reported in the studies where data was collected most recently may reflect that obesity is receiving increasing attention from the media [37], government organisations [38], and from research institutions. This may have increased awareness of the obesity epidemic in the Irish population and acted as a disincentive for obese children and their parents to participate in studies measuring BMI. Alternatively, the lower prevalence of morbid obesity in the most recent study may reflect a small downward shift in the population distribution of BMI in children in the Irish population [39].

To date, few childhood obesity interventions have been implemented in the ROI and interventions are unlikely to explain why childhood overweight and obesity rates may be stabilising. Recent interventions in the ROI have targeted specific populations such as those who are morbidly obese [40]. Other interventions have targeted specific behaviours associated with obesity including fruit and vegetable consumption [41], physical activity levels [42] or screen time [32]. The magnitude of the problem of childhood overweight and obesity in the ROI requires interventions which should be targeted at a population level. Other explanations for our findings include the relatively short time frame of included studies. A greater time period may be required to observe a clear trend in prevalence rates, especially when comparing studies with different sample sizes, age ranges and using varying methods.

### Childhood overweight and obesity rates in other developed countries

Though the prevalence of childhood obesity appears to have stabilised in a number of countries, the prevalence of overweight and obesity continues to vary significantly between and within countries. The current prevalence of overweight and obesity in the ROI is broadly similar to other European estimates. For example, the ENERGY-Project study measured BMI across seven European countries and found that 25.8% of boys and 21.8% of girls were overweight or obese though prevalence rates did vary from 14% in girls from Belgium to 44% of boys from Greece [43]. However, the findings of this review suggest that the prevalence of overweight and obesity in the ROI is higher in girls than boys. Social and economic factors may help explain why prevalence rates vary between countries. Brug et al. 2012, suggest that socio-economic factors or cultural factors may play an important role when explaining varying overweight and obesity prevalence rates between countries [44].

### Monitoring of overweight and obesity prevalence rates

Monitoring childhood obesity prevalence rates is an important public health measure. In the ROI, trends in childhood overweight and obesity had not been routinely monitored prior to the introduction of the WHO European Childhood Obesity Surveillance programme in 2008. Three phases of WHO surveillance data have now been collected in 2008, 2010 and 2012 [13]. Over time, this data will create a national database which will be comparable to surveillance data collected in other European counties [45].

All children in senior infants (year two of enrolment) in primary schools in the ROI receive a health check. Measurement of height and weight is to be included in a small subsample of schools. Based on this pilot project, height and weight may be added to this routine health check. This would provide valuable information on the height and weight of Irish children. However, ongoing surveillance initiatives do not reduce the value of other studies collecting objective height and weight data though it is essential that methods used between studies are standardised.

### Recommendations for study reporting

This review has resulted in two recommendations for study reporting. Firstly, confidence intervals or standard errors should be reported with prevalence estimates. This did not commonly occur in the included studies. Secondly, studies should provide sufficient detail which would allow for replication of the methods used.

### Strengths and limitations

A comprehensive search strategy was used to locate relevant literature and contact with obesity experts in Ireland resulted in some additional studies being identified. A critical appraisal tool was adapted to assess the quality and potential sources of bias within each included study. However, a standard critical appraisal tool to access the quality of studies reporting prevalence estimates needs to be developed. This review also has a number of limitations. The interpretation of the findings of this review was difficult due to varying methods used in the included studies. As detailed above, few of the included studies reported confidence intervals or standard errors. It was therefore difficult to interpret the accuracy of the point estimates.

## Conclusion

Though this review includes studies from a relatively short, 10 year time frame, the prevalence of overweight and obesity in school aged children in the ROI appears to be stabilising. In the absence of routinely measured data from large and representative population samples, we urge caution in the interpretation of these findings. There is a clear need to agree and disseminate standardised operating procedures and methods for the conduct of studies on the prevalence of overweight and obesity in childhood with particular reference to the issues of sampling and response rates. Although the findings provide some grounds for cautious optimism, one in four Irish children remains overweight or obese. Thus, it is clear that childhood overweight and obesity will remain an urgent priority issue for public policy for the foreseeable future.

## Electronic supplementary material

Additional file 1: **Supplementary information on the search strategy for a systematic review.** (PDF 494 KB)

Additional file 2: **Supplementary information on critical appraisals for a systematic review.** (DOCX 40 KB)

## Abbreviations

BMI: Body mass index
COSI: Childhood obesity surveillance initiative
HSE: Health Service Executive
IOTF: International Obesity Taskforce
PPS: Probability proportionate to size
ROI: Republic of Ireland
WHO: World Health Organization.
