# Supplementary material for: Trends and prevalence of overweight and obesity in primary school aged children in the Republic of Ireland from 2002-2012: a systematic review
Source: BMC Public Health. 2014 Oct 14;14:974. doi: 10.1186/1471-2458-14-974 (PMC4197331; doi:10.1186/1471-2458-14-974)
Supplement: Supplementary file 1 — Additional file 1: Supplementary information on the search strategy for a systematic review. (PDF 494 KB) [file 12889_2014_7148_MOESM1_ESM.pdf]

Supplementary information on the search strategy for a systematic review

Title

Trends and prevalence of overweight and obesity in primary school aged children in the Republic of Ireland from 2002-2012: a systematic review

Authors

Ms. Eimear Keane

Prof. Patricia M Kearney

Prof. Ivan J Perry

Prof. Cecily C Kelleher

Dr. Janas M Harrington

ADDITIONAL FILE 1A: SEARCH STRATEGY FOR MEDLINE (SEARCHED UP TO AND INCLUDING 31 MAY 2013)

1. Obesity (MeSH)

2. Overweight (MeSH)

3. Obese

4. Body Mass Index (MeSH)

5. BMI

6. Child (MeSH)

7. Child*

8. School children

9. Schoolchildren

10. Pediatr*

11. Paediatr*

12. Boys

13. Girls

14. Prevalence (MeSH)

15. Rate

16. Trend

17. Increase

18. Decrease

19. Ireland (MeSH)

20. Irish

21. 1 OR 2 OR 3 OR 4 OR 5

22. 6 OR 7 OR 8 OR 9 OR 10 OR 11 OR 12 OR 13

23. 14 OR 15 OR 16 OR 17 OR 18

24. 19 OR 20

25. 21 OR 22 OR 23 OR 24 (Limits: date of publication: 20020101-20130631; Age related: Child, Preschool: 2-5 years, Child: 6-12 years)

Results: 19

ADDITIONAL FILE 1B: SEARCH STRATEGY FOR CINAHL (SEARCHED UP TO AND INCLUDING 31 MAY 2013)

1. Obesity (MeSH)

2. Overweight

3. Obese

4. BMI

5. Body Mass Index (MeSH)

6. Schoolchildren

7. School children

8. Child*

9. Pediatr*

10. Paediatr*

11. Boys

12. Girls

13. Prevalence (MeSH)

14. Rate

15. Trend

16. Increase

17. Decrease

18. Ireland (MeSH)

19. Irish

20. 1 OR 2 OR 3 OR 4 OR 5

21. 6 OR 7 OR 8 OR 9 OR 10 OR 11 OR 12

22. 13 OR 15 OR 16 OR 17 OR 17

23. 18 OR19

24. 20 OR 21 OR 22 OR 23 (Limits: Age related: Child, Preschool: 2-5 years, Child: 6-12 years)

Results: 4

ADDITIONAL FILE 1C: SEARCH STRATEGY FOR EMBASE (SEARCHED UP TO AND INCLUDING 31 MAY 2013)

1. 'Obesity' /exp OR obesity

2. 'Overweight'/ exp OR overweight

3. Obese

4. BMI

5. 'Body Mass Index'/exp OR 'body mass index'

6. 'Children'/ exp OR children

7. 'Child'/exp OR child

8. 'Schoolchildren'/ exp OR schoolchildren

9. 'School children'/ exp OR 'school children'

10. Pediatr*

11. Paediatr*

12. Boys

13. Girls

14. 'Prevalence'/exp OR prevalence

15. Rate

16. Trend

17. Increase

18. Decrease

19. 'Ireland'/exp OR ireland

20. Irish

21. 1 OR 2 OR 3 OR 4 OR 5

22. 6 OR 7 OR 8 OR 9 OR 10 OR 11 OR 12 OR 13

23. 14 OR 15 OR 16 OR 17 OR 18

24. 19 OR 20

25. 21 OR 22 OR 23 OR 24 (Limits: ([school]/lim OR [child]/lim) AND [2002-2013]/py

Results: 285

ADDITIONAL FILE 1D: SEARCH STRATEGY FOR ACADEMIC SEARCH COMPLETE (SEARCHED UP TO AND INCLUDING 31 MAY 2013)

1. Obesity

2. Overweight

3. Obese

4. BMI

5. Body Mass Index

6. Child*

7. Schoolchildren

8. School children

9. Pediatr*

10. Paediatr*

11. Boys

12. Girls

13. Prevalence

14. Rate

15. Trend

16. Increase

17. Decrease

18. Ireland

29. Irish

20. 1 OR 2 OR 3 OR 4 OR 5

21. 6 OR 7 OR 8 OR 9 OR 10 OR 11 OR 12

22. 13 OR 14 OR 15 OR 16 OR 17

23. 18 OR 19

24. 20 OR 21 OR 22 OR 23 (Limits: date of publication: 20020101-20130631)

Results: 227

ADDITIONAL FILE 1E: SEARCH OF 'OTHER' SOURCES

Source	Methods	Results
**Source 1**<br>Authors of this systematic reviews had knowledge of potentially relevant data sources	The authors of this review were aware of grey literature potentially relevant to this systematic review	2 potentially relevant sources located
**Source 2**<br>Google search on www.google.ie (searched on 31 May 2013)	Search terms used: prevalence, child, obesity, Ireland<br>Limits: .ie websites searched only<br>The first 20 pages were searched only	1 potentially relevant source located
**Source 3**<br>Publically available Irish databases or national agencies websites (searched in April and May 2013)	Websites searched:<br>(1) Irish Social Science Data Archive (http://www.ucd.ie/issda/)<br>(2) Safefood (http://www.safefood.eu/Home)<br>(3) The Health Well (http://www.thehealthwell.info/)<br>(4) Department of Health and Children's Irish child health database (http://www.childrensdatabase.ie/irish-child-health-database/)	0 potentially relevant sources located
**Source 4**<br>Contact with obesity experts in Ireland	1. An announcement was made at an Irish obesity action forum meeting held in June 2013 (http://www.safefood.eu/Professional/Nutrition/All-island-Obesity-Action-Forum.aspx)<br>2. An email was sent to 6 obesity experts in Ireland. All 6 experts replied and many forwarded the original email to colleagues	7 potentially relevant sources located
**Source 5**<br>Reference searching of included papers	The reference section of each included paper was assessed for potentially relevant literature	1 potentially relevant source located
		11 potentially relevant source located

Footnote: Potentially relevant literature had titles/abstracts screened and if relevant the full document was read for inclusion in the systematic review.

ADDITIONAL FILE 1F: TABLE OF EXCLUDED STUDIES

Study	Detail
Griffin et al., 2004 [1]	Data for study was collected prior to 2002
Coulter & Woods, 2011[2]	No prevalence estimates available
Murphy, 2005 [3]	No prevalence estimates available
Finucane, 2009 [4]	No prevalence estimates available

REFERENCES

1. Griffin AC, Younger KM, Flynn MAT: **Assessment of obesity and fear of fatness among inner-city Dublin schoolchildren in a one-year follow-up study**. *Public health nutrition* 2004, **7**(6):729-735.
2. Coulter M, Woods CB: **An exploration of children's perceptions and enjoyment of school-based physical activity and physical education**. *Journal of physical activity & health* 2011, **8**(5):645-654.
3. Murphy JFA: **The National Taskforce on Obesity**. *Irish Medical Journal* 2005, **98**(5):132-132.
4. Finucane F: **Obesity in Irish youth: epidemiology and implications**. *Irish Journal of Medical Science* 2009, **178**(3):249-255.
