# Supplementary material for: Trends and prevalence of overweight and obesity in primary school aged children in the Republic of Ireland from 2002-2012: a systematic review
Source: BMC Public Health. 2014 Oct 14;14:974. doi: 10.1186/1471-2458-14-974 (PMC4197331; doi:10.1186/1471-2458-14-974)
Supplement: Supplementary file 2 — Additional file 2: Supplementary information on critical appraisals for a systematic review. (DOCX 40 KB) [file 12889_2014_7148_MOESM2_ESM.docx]

**Supplementary information on critical appraisals for a systematic review**

**Title**

Trends and prevalence of overweight and obesity in primary school aged children in the Republic of Ireland from 2002-2012: a systematic review

**Authors**

Ms. Eimear Keane

Prof. Patricia M Kearney

Prof. Ivan J Perry

Prof. Cecily C Kelleher

Dr. Janas M Harrington

**Quality assessment checklist used:**

|  | **Methodological criteria** | **Criterion adequate if:** | **Scored 1 if adequate** |
| --- | --- | --- | --- |
| 1 | Was the target population specified? | Information was outlined on the age of participants to be recruited and the geographical location of study |  |
| 2 | Was a sampling method employed?  Was the sample random, non-random or a complete sample? Is the study population representative of the target population? | The sampling method was defined clearly |  |
| 3 | Was the response rate (RR) adequate? | The RR was outlined and if greater than 70% of participants at the final sampling unit participated in the study |  |
| 4 | Was information given on non-responders? | Information on anthropometric indicators was provided on those who did not participate in the study |  |
| 5 | Were the cut-off points used to define overweight and obesity specified? | The cut-off points used were outlined in the paper/report |  |
| 6 | Were details on the method of the height and weight measurements specified in adequate detail? | The method of height and weight were outlined in detail where replication of the methods would be possible |  |
| 7 | Were details on the height and weight instruments used specified? Was the equipment calibrated? | The name of the height and weight instruments were specified |  |
| 8 | Was an effort made to reduce observer bias? Did a trained researcher taken the measurements? **OR** Were statistical methods used to access observer bias? | Methods to reduce observer bias prior to and/or during fieldwork were outlined such as training or re-training |  |
|  |  |  | /8 |

**Author: Whelton et al., 2007 [1, 2]**

|  | **Methodological criteria** | **Data extracted** |  |
| --- | --- | --- | --- |
| 1 | Was the target population specified? | Children and adolescents of a school going age (4-16*) from schools in the Republic of Ireland (ROI) and Northern Ireland were eligible to participate | 1 |
| 2 | Was a sampling method employed? Was the sample random, non-random or a complete sample?  Is the study population representative of the target population? | A cluster sampling method was employed with the school as the primary sampling unit. Children from junior infants, 2^nd^ class and 6^th^ class from primary schools (year 1, 4 and 8 of enrolment respectively) were randomly selected to participate on the basis of age, sex, location and water supply type (fluoridated or not) of the school attended  Yes | 1 |
| 3 | Was the RR adequate? | 68% of children in the ROI participated | 0 |
| 4 | Was information given on non-responders? | No | 0 |
| 5 | Were the cut-off points used to define overweight and obesity specified? | International Obesity Taskforce (IOTF) cut-off points were used | 1 |
| 6 | Were details on the method of the height and weight measurements specified in adequate detail? | Height was measured to the nearest 1 decimal point in centimetre (cm) and weight to the nearest 1 decimal point in kilograms (kg) without shoes, heavy clothing and headgear | 1 |
| 7 | Were details on the height and weight instruments used specified? Was the equipment calibrated? | A Leicester height measure and a Soehnle 7403 Mediscale weight scales were used  The weight scales were calibrated before and after fieldwork | 1 |
| 8 | Was an effort made to reduce observer bias? Did a trained researcher taken the measurements? Were statistical methods used to access observer bias? | Standard equipment was used. The research team were trained and a validation exercise was conducted during fieldwork  No statistics were used to assess observer bias | 1 |
|  |  |  | 6/8 |

**Footnote**: *The figures presented in this systematic review exclude secondary school children and children from Northern Ireland

**Author: O’Neill et al., 2006 [3]**

|  | **Methodological criteria** | **Data extracted** |  |
| --- | --- | --- | --- |
| 1 | Was the target population specified? | A nationally representative sample of Irish children (aged 5-12 years) were sampled from primary schools in the ROI | 1 |
| 2 | Was a sampling method employed? Was the sample random, non-random or a complete sample?  Is the study population representative of the target population? | A list of primary schools was obtained from the Department of Education and Science. Schools were classified by school size, gender, disadvantaged status and location. Schools were randomly selected from each category and children were randomly sampled from within each school  Yes | 1 |
| 3 | Was the RR adequate? | Over 90% of schools and 66% of children took part | 0 |
| 4 | Was information given on non-responders? | No | 0 |
| 5 | Were the cut-off points used to define overweight and obesity specified? | Prevalence rates are presented using four different definitions including the IOTF definitions which we present in this systematic review | 1 |
| 6 | Were details on the method of the height and weight measurements specified in adequate detail? | Height was measured in the Frankfurt plane to the nearest 0.1cm and weight was measured to the nearest 0.1kg. Measurements were taken without shoes, in light clothing and after voiding. Weight was measured in duplicate | 1 |
| 7 | Were details on the height and weight instruments used specified? Was the equipment calibrated? | A SECA Leicester height measure and a SECA 770 digital weight scales were used  No details provided on the calibration of equipment | 1 |
| 8 | Was an effort made to reduce observer bias? Did a trained researcher taken the measurements? Were statistical methods used to access observer bias? | Qualified nutritionists took measures though it is not stated if they were provided with training  No statistics were used to assess observer bias | 0 |
|  |  |  | 5/8 |

**Author: Layte & McCrory, 2011 [4]**

|  | **Methodological criteria** | **Data extracted** |  |
| --- | --- | --- | --- |
| 1 | Was the target population specified? | A nationally representative sample of 9 year old children residing in the ROI | 1 |
| 2 | Was a sampling method employed? Was the sample random, non-random or a complete sample?  Is the study population representative of the target population? | A two stage sampling process was used with primary schools as the primary sampling unit and children as the second sampling unit. A probability proportionate to size (PPS) sampling strategy was used to sample primary schools and a random sample of age eligible children were selected from within participating schools  Yes | 1 |
| 3 | Was the RR adequate? | No, 82% of schools and 57% of children participated in the study | 0 |
| 4 | Was information given on non-responders? | No. The data was probability weighted prior to analysis to ensure finding were nationally representative | 1 |
| 5 | Were the cut-off points used to define overweight and obesity specified? | IOTF cut-off points were used | 1 |
| 6 | Were details on the method of the height and weight measurements specified in adequate detail? | Height was measured to the nearest millimetre (mm) and weight to the nearest 0.5kg. Children wore light clothes for the measurements | 1 |
| 7 | Were details on the height and weight instruments used specified? Was the equipment calibrated? | A Leicester height measure and a SECA 761 flat mechanic weight scales were used  No details provided on the calibration of equipment | 1 |
| 8 | Was an effort made to reduce observer bias? Did a trained researcher taken the measurements? Were statistical methods used to access observer bias? | Researchers were trained  No statistics were used to assess observer bias | 1 |
|  |  |  | 7/8 |

**Author: Heavey et al., 2009 [5]**

|  | **Methodological criteria** | **Data extracted** |  |
| --- | --- | --- | --- |
| 1 | Was the target population specified? | This study is part of the World Health Organization (WHO) European Childhood Obesity Surveillance programme. Seven year old children living in the ROI were the target population | 1 |
| 2 | Was a sampling method employed? Was the sample random, non-random or a complete sample?  Is the study population representative of the target population? | A PPS sampling strategy was used to sample schools. Children in first class (year 3 of enrolment) were invited to partake. In larger schools one class was selected to partake and in smaller schools, all available age eligible children were invited to partake. Small schools are underrepresented in final sample due to logistical reasons  Yes | 1 |
| 3 | Was the RR adequate? | Yes, 33% of small schools and 48% of large schools took part and 72% of children participated | 1 |
| 4 | Was information given on non-responders? | No. Those without consent forms and those not present at school on the day of measurements were excluded | 1 |
| 5 | Were the cut-off points used to define overweight and obesity specified? | IOTF cut-off points were used | 1 |
| 6 | Were details on the method of the height and weight measurements specified in adequate detail? | Height was measured to the last complete mm and weight to the nearest 0.1kg. Children wore light indoor clothing, no shoes, hair ornaments were removed, pony tails undone and pockets were emptied for measures | 1 |
| 7 | Were details on the height and weight instruments used specified? Was the equipment calibrated? | A SECA 214 portable stadiometer and a SECA 872 weighing scales were used  Equipment was calibrated prior to study commencing | 1 |
| 8 | Was an effort made to reduce observer bias? Did a trained researcher taken the measurements? Were statistical methods used to access observer bias? | Thirty nutritionists/study researchers were trained in anthropometry prior to the study commencing. A standard operating procedure (SOP) drawn up by WHO was adhered to during the study  No statistics were used to assess observer bias | 1 |
|  |  |  | 7/8 |

**Author: Heinen et al., 2014 [6]**

|  | **Methodological criteria** | **Data extracted** |  |
| --- | --- | --- | --- |
| 1 | Was the target population specified? | This study is part of the WHO European Childhood Obesity Surveillance programme. In round 2, first and third class children (years 3 and 5 of enrolment into primary school) were recruited. In round 3, first, third and fifth class children (years 3, 5 and 7 of enrolment in primary schools) were recruited* | 1 |
| 2 | Was a sampling method employed? Was the sample random, non-random or a complete sample?  Is the study population representative of the target population? | The same schools which participated in round one of data collection in 2008 were approached to participate in rounds 2 and 3.  In larger schools one class was selected to partake and in smaller schools, all available age eligible children were invited to partake  Yes | 1 |
| 3 | Was the RR adequate? | At round 2, at a school level 81.0% of first classes and 64.2% of first class children participated in the study. At round 3, at a school level 82.4% of first classes and 54.8% of first class children took part in the study | 0 |
| 4 | Was information given on non-responders? | No. Those without consent forms and those not present at school on the day of measurements were excluded | 0 |
| 5 | Were the cut-off points used to define overweight and obesity specified? | IOTF cut-off points were used | 1 |
| 6 | Were details on the method of the height and weight measurements specified in adequate detail? | Height was measured to the last complete mm and weight to the nearest 0.1kg. Children wore light indoor clothing, no shoes, hair ornaments were removed, pony tails undone and pockets were emptied for measurements | 1 |
| 7 | Were details on the height and weight instruments used specified? Was the equipment calibrated? | A SECA 214 portable stadiometer and a SECA 872 weighing scales were used at round 2 and a HD-305 Tanita weighing scales and a Leicester Height measure were used at round 3. Equipment was calibrated prior to study (round 2 and round 3) commencing | 1 |
| 8 | Was an effort made to reduce observer bias? Did a trained researcher taken the measurements? Were statistical methods used to access observer bias? | Fifteen and 17 nutritionists/study researchers (at rounds 2 and 3) were trained in anthropometry prior to the study commencing. An SOP drawn up by WHO was adhered to during the study  No statistics were used to assess observer bias | 1 |
|  |  |  | 6/8 |

**Footnote:** *For the purposes of this systematic review, we only included prevalence rates from children in first class (year 3 of enrolment)

**Author: McMaster et al., 2005 [7]**

|  | **Methodological criteria** | **Data extracted** |  |
| --- | --- | --- | --- |
| 1 | Was the target population specified? | Children from senior infants(year two of enrolment) in primary schools located in the former North West Health Board area in County Leitrim and part of Count Cavan | 1 |
| 2 | Was a sampling method employed? Was the sample random, non-random or a complete sample?  Is the study population representative of the target population? | A complete sample of schools and senior infants within this former North West Health Board area. Children were in senior infants in 2001/2002  Yes | 1 |
| 3 | Was the RR adequate? | Hand searching of file resulted in 361records being located. This represents 98% of children from schools in this area based on Department of Education records. Overall, 328 (91%) of children had height and weight measurements available | 1 |
| 4 | Was information given on non-responders? | No. Children without consent, without follow-up or those attending a special school did not have measurements taken | 0 |
| 5 | Were the cut-off points used to define overweight and obesity specified? | Prevalence rates are presented using two different definitions including the IOTF definitions which we present in this systematic review | 1 |
| 6 | Were details on the method of the height and weight measurements specified in adequate detail? | Height was measured to the nearest 0.5cm and weight to the nearest 500g in light clothing, without shoes, jackets and headgear. A regionally agreed measurement protocol was followed | 1 |
| 7 | Were details on the height and weight instruments used specified? Was the equipment calibrated? | A Leicester height measure and a Hansen Digital weight scales were used  The weight scales were calibrated yearly | 1 |
| 8 | Was an effort made to reduce observer bias? Did a trained researcher taken the measurements? Were statistical methods used to access observer bias? | Two school nurses took measurements while adhering to regionally agreed protocol and using standard equipment  No statistics were used to assess observer bias | 1 |
|  |  |  | 7/8 |

**Author: Harrison et al., 2006 [8]**

|  | **Methodological criteria** | **Data extracted** |  |
| --- | --- | --- | --- |
| 1 | Was the target population specified? | Fourth class children (year 6 of enrolment) attending primary schools located in areas of social disadvantage in the South East of the ROI | 1 |
| 2 | Was a sampling method employed? Was the sample random, non-random or a complete sample?  Is the study population representative of the target population? | Unsure of sampling method employed. Convenience sampling may have been used. Nine schools were recruited and all fourth class children invited to participate  Unsure | 0 |
| 3 | Was the RR adequate? | Yes, 99% of invited children participated | 1 |
| 4 | Was information given on non-responders? | No. One percent of children did not have parental consent to participate | 0 |
| 5 | Were the cut-off points used to define overweight and obesity specified? | IOTF cut-off points were used | 1 |
| 6 | Were details on the method of the height and weight measurements specified in adequate detail? | Height and weight were measured without shoes and without heavy clothing. No other details given regarding measurement units used | 0 |
| 7 | Were details on the height and weight instruments used specified? Was the equipment calibrated? | A Seca Leicester height measure and a SECA digital floor scales were used  No details provided on the calibration of equipment | 1 |
| 8 | Was an effort made to reduce observer bias? Did a trained researcher taken the measurements? Were statistical methods used to access observer bias? | Researchers were trained in anthropometry  No statistics were used to assess observer bias | 1 |
|  |  |  | 5/8 |

**Author: Evans et al., 2010 [9]**

|  | **Methodological criteria** | **Extracted data** |  |
| --- | --- | --- | --- |
| 1 | Was the target population specified? | All children in senior infants (year 2 of enrolment) in all primary schools in County Mayo between February 2005 and June 2008. | 1 |
| 2 | Was a sampling method employed? Was the sample random, non-random or a complete sample?  Is the study population representative of the target population? | A complete sample (N=189) of schools and all senior infants within each school. In 2005, 74 schools took part as all other schools had the school heath check complete*prior to this study commencing  Yes | 1 |
| 3 | Was the RR adequate? | Overall, 99.7% of children took part | 1 |
| 4 | Was information given on non-responders? | No. Non-responders were either absent from school (N=5), unable to stand independently (N=5) or had no parental consent (N=6) | 0 |
| 5 | Were the cut-off points used to define overweight and obesity specified? | IOTF cut-off points were used | 1 |
| 6 | Were details on the method of the height and weight measurements specified in adequate detail? | Height was measured to the nearest 0.1cm and weight to the nearest 0.1kg. A study protocol was adhered to during measurements | 1 |
| 7 | Were details on the height and weight instruments used specified? Was the equipment calibrated? | A Tanita Solar portable weighing scales and a Leicester height measure were used  Equipment was calibrated | 1 |
| 8 | Was an effort made to reduce observer bias? Did a trained researcher taken the measurements? Were statistical methods used to access observer bias? | Five public health nurses took the measures.  Training was undertaken and specified guidelines were adhered to  Intra-observer variability was measured by repeating every 10^th^ measure. Kendall’s tau b was used to determine the intra-observer agreement | 1 |
|  |  |  | 7/8 |

**Footnote:*** The measurement of height and weight was included as part of the school health check. Height and weight are not routinely measured as part of the school health check but were included for the purposes of this study

**Author: Barron et al., 2009 [10]**

|  | **Methodological criteria** | **Extracted data** |  |
| --- | --- | --- | --- |
| 1 | Was the target population specified? | Primary school children (aged 4-13 years) from one town in Co. Kildare | 1 |
| 2 | Was a sampling method employed? Was the sample random, non-random or a complete sample?  Is the study population representative of the target population? | Not stated. Two schools in a town in County Kildare took part. All classes in both schools were invited to participate. The height and weight data was collected as part of a larger study  Unsure | 0 |
| 3 | Was the RR adequate? | 83% of children in the participating schools (N=2) participated | 1 |
| 4 | Was information given on non-responders? | No. Those with medical conditions affecting growth were excluded. Those who did not receive parental consent/ not willing to participate did not have measurements taken | 0 |
| 5 | Were the cut-off points used to define overweight and obesity specified? | IOTF cut-off points were used | 1 |
| 6 | Were details on the method of the height and weight measurements specified in adequate detail? | Children were weighed in tracksuits, without shoes. No other details given regarding measurement units used | 0 |
| 7 | Were details on the height and weight instruments used specified? Was the equipment calibrated? | A Tanita WB-100 digital weighing scales and a Leicester height measure were used  No details provided on the calibration of equipment | 1 |
| 8 | Was an effort made to reduce observer bias? Did a trained researcher taken the measurements? Were statistical methods used to access observer bias? | One paediatric nurse took all measures | 1 |
|  |  |  | 5/8 |

**Footnote:** Lead author contacted to clarify details on methodological criteria 2

**Author: Murrin et al., 2012 [11, 12]**

|  | **Methodological criteria** | **Extracted data** |  |
| --- | --- | --- | --- |
| 1 | Was the target population specified? | Expectant mothers at their first maternity hospital booking visit from two representative regions recruited during an 18 month period in 2001-2003. Hospital were from one urban (Coombe Women’s Hospital, Dublin) and one rural (University College Hospital, Galway) area. Mother’s born outside Ireland were excluded | 1 |
| 2 | Was a sampling method employed? Was the sample random, non-random or a complete sample?  Is the study population representative of the target population? | Non-random consecutive sampling used. Not all women were approached due to staffing levels in the hospital  Yes | 1 |
| 3 | Was the RR adequate? | No RR given for phase one (recruitment stage) though 62% of parents responded at follow up when children were aged 5 years (N=669) and 529 children were included in the analysis | 0 |
| 4 | Was information given on non-responders? | Yes, those who could not be followed up did not have significantly different BMIs at baseline versus responders | 1 |
| 5 | Were the cut-off points used to define overweight and obesity specified? | IOTF cut-off points were used | 1 |
| 6 | Were details on the method of the height and weight measurements specified in adequate detail? | Height was measured to the nearest 1cm and weight to the nearest 0.1kg. A protocol was adhered to during measurements | 1 |
| 7 | Were details on the height and weight instruments used specified? Was the equipment calibrated? | A Tanita digital weighing scales HD305A and a Leicester height measure were used  Equipment was calibrated | 1 |
| 8 | Was an effort made to reduce observer bias? Did a trained researcher taken the measurements? Were statistical methods used to access observer bias? | Researchers were trained and a standard protocol used  No statistics were used to assess observer bias | 1 |
|  |  |  | 7/8 |

**Author: Belton et al., 2010 [13]**

|  | **Methodological criteria** | **Data extracted** |  |
| --- | --- | --- | --- |
| 1 | Was the target population specified? | Irish primary school children in 2^nd^ and 3^rd^ classes (year 4 and 5 of enrolment) from the greater Dublin area | 1 |
| 2 | Was a sampling method employed? Was the sample random, non-random or a complete sample?  Is the study population representative of the target population? | Sampling strategy unclear. Four schools participated in the study  Unsure | 0 |
| 3 | Was the RR adequate? | 301 of 311 (97%) children took part | 1 |
| 4 | Was information given on non-responders? | No. Those without parental consent did not take part in the study | 0 |
| 5 | Were the cut-off points used to define overweight and obesity specified? | IOTF cut-off points were used | 1 |
| 6 | Were details on the method of the height and weight measurements specified in adequate detail? | Methods of measurements were not specified | 0 |
| 7 | Were details on the height and weight instruments used specified? Was the equipment calibrated? | A SECA Leicester height measure and a SECA heavy duty weight scales (specific scales type not specified) were used  No details provided on the calibration of equipment | 0 |
| 8 | Was an effort made to reduce observer bias? Did a trained researcher taken the measurements? Were statistical methods used to access observer bias? | Methods to reduce observer bias not specified in paper  No statistics were used to assess observer bias | 0 |
|  |  |  | 3/8 |

**Author: Fitzgerald, 2010 [14]**

|  | **Methodological criteria** | **Data extracted** |  |
| --- | --- | --- | --- |
| 1 | Was the target population specified? | Children attending primary and post primary schools in the West of Ireland. Children aged at least 9 years of age* | 1 |
| 2 | Was a sampling method employed? Was the sample random, non-random or a complete sample?  Is the study population representative of the target population? | A random sample of schools were selected from a Department of Education and Science list of schools. Schools were located in 3 counties in the West of Ireland  Yes | 1 |
| 3 | Was the RR adequate? | No, 58% of children invited took part | 0 |
| 4 | Was information given on non-responders? | No. Children without parental consent did not take part | 0 |
| 5 | Were the cut-off points used to define overweight and obesity specified? | IOTF cut-off points were used | 1 |
| 6 | Were details on the method of the height and weight measurements specified in adequate detail? | Weight was measured to the nearest 0.1kg and height to the nearest 0.1cm in the Frankfurt plane position. Measurements were taken without shoes and without heavy clothing | 1 |
| 7 | Were details on the height and weight instruments used specified? Was the equipment calibrated? | A Seca 899 weight scales and a Leicester height measure were used  No details provided on the calibration of equipment | 1 |
| 8 | Was an effort made to reduce observer bias? Did a trained researcher taken the measurements? Were statistical methods used to access observer bias? | Standard procedures were used. Intra-observer variability was tested by repeating every 10^th^ measurement  No statistics were used to assess observer bias | 1 |
|  |  |  | 6/8 |

**Footnote:** *Only primary school children are included in this systematic review. This is unpublished data. Information was obtained from the study protocol and from details provided by a lead researcher of the study

**Author: HSE Meath, unpublished observation**

|  | **Methodological criteria** | **Data extracted** |  |
| --- | --- | --- | --- |
| 1 | Was the target population specified? | All 6^th^ class (year 8 of enrolment) children from a complete sample of primary schools in County Meath | 1 |
| 2 | Was a sampling method employed? Was the sample random, non-random or a complete sample?  Is the study population representative of the target population? | A complete sample of primary schools in County Meath were invited to participate. All children in 6^th^ class from participating schools were invited to take part  Yes | 1 |
| 3 | Was the RR adequate? | School RR not specified but 63% of children took part in the study | 0 |
| 4 | Was information given on non-responders? | No | 0 |
| 5 | Were the cut-off points used to define overweight and obesity specified? | IOTF cut-off points were used | 1 |
| 6 | Were details on the method of the height and weight measurements specified in adequate detail? | Weight in kg and height in cm were recorded to one decimal point. Measurements were taken without shoes and without excessive clothing. Height was measured in the Frankfurt plane position | 1 |
| 7 | Were details on the height and weight instruments used specified? Was the equipment calibrated? | A c Soehnle 7403 Mediscale and a Leicester Height measure were used  Equipment was calibrated | 1 |
| 8 | Was an effort made to reduce observer bias? Did a trained researcher taken the measurements? Were statistical methods used to access observer bias? | Inter examiner agreement was tested by having both examiners visit one school  No statistics were used to assess observer bias | 1 |
|  |  |  | 6/8 |

**Footnote:** This is unpublished data. Information was obtained from the study protocol and details on response rate were provided by a lead researcher of the study

**Author: Hollywood et al., 2012 [15]**

|  | **Methodological criteria** | **Data extracted** |  |
| --- | --- | --- | --- |
| 1 | Was the target population specified? | Children aged 4-12 years from schools in disadvantaged areas of Dublin (school located in Revitalising Areas by Planning Investment and Development [RAPID] areas) | 1 |
| 2 | Was a sampling method employed? Was the sample random, non-random or a complete sample?  Is the study population representative of the target population? | Seven schools located in RAPID areas in Dublin. Author of paper was contacted, the organisation who ‘rolled out’ the Healthy Schools Programme chose the intervention schools and the comparison schools) were chosen by the study research team  Unsure | 1 |
| 3 | Was the RR adequate? | Over 50% of children from participating schools took part in the study | 0 |
| 4 | Was information given on non-responders? | No. Those without consent, who did not want to take part on the day of the study and those in 6^th^ class (year 8 of enrolment) were excluded from the baseline study | 0 |
| 5 | Were the cut-off points used to define overweight and obesity specified? | IOTF cut-off points were used | 1 |
| 6 | Were details on the method of the height and weight measurements specified in adequate detail? | Height was measured in the Frankfurt plane, without shoes and a weighted headboard not used during measurements. Weight was measured without shoes and with heavy clothing removed. No other details given regarding measurement units used | 0 |
| 7 | Were details on the height and weight instruments used specified? Was the equipment calibrated? | A SECA Leicester height measure and a SECA 875 digital flat weight scales were used  No details provided on the calibration of equipment | 1 |
| 8 | Was an effort made to reduce observer bias? Did a trained researcher taken the measurements? Were statistical methods used to access observer bias? | One children’s nurse took all the measures. This nurse received training prior to the study commencing | 1 |
|  |  |  | 5/8 |

**Footnote:** Lead author contacted to clarify details on methodological criteria 2

**Author: Keane et al., unpublished observation**

|  | **Methodological criteria** | **Data extracted** |  |
| --- | --- | --- | --- |
| 1 | Was the target population specified? | Third and fourth class (years 5 and 6 of enrolment) children in primary schools in Cork City & Mitchelstown in Cork County. | 1 |
| 2 | Was a sampling method employed? Was the sample random, non-random or a complete sample?  Is the study population representative of the target population? | Cork City schools were recruited using a PPS sampling strategy. Further purposive sampling of City schools was needed to achieve the desired sample size (N=1,000). A complete sample of schools from Mitchelstown were invited to partake. All 3^rd^ and 4^th^ class children from participating schools were invited to participate.  Slight gender imbalance but the sample is representative of target population | 1 |
| 3 | Was the RR adequate? | No, 59% of schools and 65% of children took part in the study | 0 |
| 4 | Was information given on non-responders? | No. Children without parental consent did not take part in the study | 0 |
| 5 | Were the cut-off points used to define overweight and obesity specified? | IOTF cut-off points were used | 1 |
| 6 | Were details on the method of the height and weight measurements specified in adequate detail? | Measures taken to the nearest 0.1cm and nearest 0.1kg. Measurements were taken without shoes and in light clothing | 1 |
| 7 | Were details on the height and weight instruments used specified? Was the equipment calibrated? | A Leicester height measure and a Tanita WB-100MA weight scales were used  The equipment was calibrated before the study commenced and monthly thereafter | 1 |
| 8 | Was an effort made to reduce observer bias? Did a trained researcher taken the measurements? Were statistical methods used to access observer bias? | Study researchers were trained before the study commenced. Re-training sessions were undertaken during the fieldwork period and a study protocol was adhered to during measurements  No statistics were used to assess observer bias | 1 |
|  |  |  | 6/8 |

**Footnote:** This is unpublished data. Information was obtained from the study protocol. The lead researchers of this study (EK, PMK, IJP and JMH) are co-authors of this systematic review

**REFERENCES**

1. Whelton H, Harrington J, Crowley E, Kelleher V, Cronin M, Perry IJ: **North South Survey of children's Height, Weight and Body Mass Index, 2002**. Ireland, Dublin: Department of Health and Children; 2006.

2. Whelton H, Harrington J, Crowley E, Kelleher V, Cronin M, Perry I: **Prevalence of overweight and obesity on the island of Ireland: results from the North South Survey of Children's Height, Weight and Body Mass Index, 2002**. *BMC Public Health* 2007, **7**(1):187.

3. O'Neill J, McCarthy S, Burke S, Hannon E, Kiely M, Flynn A, Flynn M, Gibney M: **Prevalence of overweight and obesity in Irish school children, using four different definitions**. *European Journal of Clinical Nutrition* 2006, **61**(6):743-751.

4. Layte R, McCrory C: **Growing Up in Ireland–Overweight and Obesity among 9-year-olds**. Ireland, Dublin: Department of Children and Youth Affairs; 2011.

5. Heavey P, McGloin A, Kilroe J, Daly L, O'Mahony D, Kelleher C: **Childhood Obesity Surveillance Initiative in Ireland. Main Report.** Ireland, Dublin: Health Service Executive and Department of Health and Children; 2009.

6. Heinen MM, Murrin C, Daly L, O'Brien J, Heavey P, Kilroe J, O'Brien M, Scully H, Mulhern LM, Lynam A, *Hayes C, O’Dwyer U, Eldin N, Kelleher C*: *The Childhood Obesity Surveillance Initiative (COSI) in the Republic of Ireland: Findings from 2008, 2010 and 2012.* Ireland, Dublin: Health Service Executive; 2014.

7. McMaster C, Cullen L, Raymond N: **Overweight and obesity in Irish primary schools: Retrospective cohort study**. *Child: Care, Health and Development* 2005, **31**(5):499-506.

8. Harrison M, Burns CF, McGuinness M, Heslin J, Murphy NM: **Influence of a health education intervention on physical activity and screen time in primary school children: 'Switch Off-Get Active'**. *Journal of Science and Medicine in Sport* 2006, **9**(5):388-394.

9. Evans DS, Glacken M, Goggin D: **Childhood obesity: the extent of the problem among 6-year-old Irish national school children**. *Child: care, health and development* 2011, **37**(3):352-359.

10. Barron C, Comiskey C, Saris J: **Prevalence rates and comparisons of obesity levels in Ireland**. *British journal of nursing* 2009, **18**(13):799-803.

11. Murrin CM, Kelly GE, Tremblay RE, Kelleher CC: **Body mass index and height over three generations: evidence from the Lifeways cross-generational cohort study**. *BMC Public Health* 2012, **12**(1):81.

12. O'Mahony D, Fallon U, Hannon F, Kloeckner K, Avalos G, Murphy A, Kelleher C: **The Lifeways Cross-Generation Study: design, recruitment and data management considerations**. *Irish Medical Journal* 2007, **100**(8):suppl 3.

13. Belton S, Brady P, Meegan S, Woods C: **Pedometer step count and BMI of Irish primary school children aged 6–9 years**. *Preventive medicine* 2010, **50**(4):189-192.

14. Fitzgerald A: **Psychological and social factors in predicting children's food choices: The development of a theoretical model.** *PhD thesis*. National University of Ireland, Galway; 2010.

15. Hollywood E, Comiskey C, Begley T, Snel A, O'Sullivan K, Quirke M, Wynne C: **Measuring and modelling body mass index among a cohort of urban children living with disadvantage**. *Journal of Advanced Nursing* 2012.
